# Impact of modern chemotherapy on the survival of women presenting with *de novo* metastatic breast cancer

**DOI:** 10.1186/1471-2407-12-435

**Published:** 2012-09-28

**Authors:** Sumanta K Pal, Mary Dehaven, Rebecca A Nelson, Susan Onami, JoAnn Hsu, Sarah Waliany, Laura Kruper, Joanne Mortimer

**Affiliations:** 1 Division of Genitourinary Malignancies, Department of Medical Oncology & Experimental Therapeutics, City of Hope Comprehensive Cancer Center, Duarte, CA, USA; 2 Department of Biostatistics, City of Hope Comprehensive Cancer Center, Duarte, CA, USA; 3 Department of Surgery, City of Hope Comprehensive Cancer Center, Duarte, CA, USA

**Keywords:** Chemotherapy, Endocrine therapy, Survival, Metastatic breast cancer, Stage IV

## Abstract

**Background:**

Data that directly associate utilization of novel systemic therapies with survival trends in metastatic breast cancer (MBC) are limited. In the setting of *de novo* MBC, large registry analyses cite positive temporal trends in survival, but the extent to which advances in systemic therapy have contributed to these gains is not clear.

**Methods:**

The City of Hope Cancer Registry was used to identify a consecutive series of patients with *de novo* MBC who received their first line of therapy between 1985 and 2004. Comprehensive clinicopathologic and treatment-related data were collected for each patient. Univariate analyses were conducted via Cox regression to identify factors associated with improved survival. Multivariate analysis was also conducted via Cox regression and the stepwise procedure was used to identify independent predictors of survival.

**Results:**

A total of 324 patients with *de novo* MBC were identified. After application of exclusion criteria, including the sole presence of supraclavicular node metastasis, 274 patients were retained in the analysis. The treatment-related characteristics associated with improved survival included: use of endocrine therapy (hazard ratio [HR] 0.60, 95%CI 0.47-0.77; P<0.0001), and addition of bisphosphonates (HR 0.70, 95%CI 0.52-0.96; P=0.02). However, recipients of novel cytotoxic agents (defined as drugs approved for MBC since 1994) had no improvement in survival relative to patients treated with older cytotoxic agents. On multivariate analysis, age (< 50), receipt of aromatase inhibitors, and receipt of zoledronic acid were independent predictors of survival.

**Conclusions:**

The overall survival of women with *de novo* metastatic breast cancer has improved over the past 20 years. However, the contribution of conventional cytotoxic agents to this improvement is minimal.

## Background

Over the past 30 years, the survival of women with early stage breast cancer has been prolonged [1]. In addition to earlier detection, the use of adjuvant chemotherapy and endocrine therapy following definitive surgery and radiation therapy is credited with a significant improvement in overall survival. However, these same systemic therapies that prolong the survival of women with early stage disease are only palliative in metastatic disease. For women with metastatic HER2 positive breast cancers, the addition of trastuzumab to first line chemotherapy has been shown to both prolong both the disease free and overall survivals [2]. Shortly after the FDA approved trastuzumab for metastatic breast cancer (MBC), an FDA panel was convened to review the indications for approving new agents as first-line therapy in advanced breast cancer. The committee recommended that improvement in overall survival should be demonstrated for an agent considered for the first line treatment of advanced breast cancer [3].

Since 1994, a number of agents have been shown to be active in advanced breast cancer. These include: paclitaxel, docetaxel, capecitabine, vinorelbine, gemcitabine, nab-paclitaxel, and ixabepilone [3]. The recent debate about the use of bevacizumab in breast cancer has forced us to re-evaluate the risk benefit ratio of our interventions and to question if prolongation in overall survival is a realistic benchmark for all new agents [4].

The goal of this study was to assess the impact of newer chemotherapy agents on overall survival of women with newly diagnosed de novo metastatic disease (i.e., primary distant MBC). Several other investigators have used existing databases to determine whether advances in systemic therapy have improved patient survival over different time intervals [5-10]. These studies have generally included both women who developed recurrent disease after adjuvant chemotherapy and/or endocrine therapy and patients treated with endocrine therapy. Using the City of Hope Cancer Registry, we compared the overall survival of women with *de novo* metastatic breast cancer treated between January 1, 1985 and December 31, 1994 with the overall survival of women diagnosed between January 1, 1995 and December 31, 2004 with attention to the impact of chemotherapy on disease outcome.

## Methods

### Patient selection

A consecutive series of patients with *de novo* MBC were identified from the City of Hope (COH) Cancer Registry through an IRB approved protocol. The registry houses data on all patients who receive cancer treatment at the institution. This database has been maintained for over three decades and includes patient demographic information and disease-related variables, including diagnosis, date of diagnosis, pathologic assessment, sites of metastasis, and type/modality of treatment. Disease and vital status are updated at least annually, and date of last follow-up or death is recorded. Vital status is confirmed by either cross-referencing clinician reports or the Social Security Death Index. Patients eligible for the current analysis were those with *de novo* MBC diagnosed between January 1, 1985 and December 31, 2004. For consideration in the current study, patients were required to have stage IV breast cancer as defined by the American Joint Committee on Cancer (AJCC) Cancer Staging Manual (6^th^ Ed) [9]. A relevant change in this edition was designation of ipsilateral supraclavicular node involvement as N3 disease (previously considered M1 disease). Thus, patients diagnosed with stage IV disease on the basis of ipsilateral supraclavicular node involvement alone were excluded from analyses. Medical records for each *de novo* MBC registry case was reviewed in full by the principal investigator (S.K.P) or trained associates (S.O.; S.W.) to ensure completeness and appropriateness for inclusion in the current study.

### Variables

Variables obtained from the registry included date of birth, date of diagnosis, date of death or last follow-up, estrogen receptor (ER) and progesterone receptor (PR) status, human epidermal growth factor receptor 2 (HER2) status, tumor histology, and sites of metastases. As hormone receptor status was not reported consistently by the cancer registry prior to January 1, 1995, incomplete data were supplemented through review of pathology records. Hormone receptor status was assigned in the context of institutional reference ranges for the assay utilized. HER2 status, primarily available for patients diagnosed with breast cancer since 1998, was also obtained from the pathology records when not recorded in the registry. Patients were considered HER2-positive if immunohistochemical staining for HER2 was classified as 2+ or 3+ or the HER2/*neu*:centromere 17 ratio determined by fluorescence *in situ* hybridization (FISH) was ≥ 2.2. Histology was classified as either inflammatory or non-inflammatory. Sites of metastasis were coded as liver, lung, brain, bone, supraclavicular node, and soft tissue. Thorough chart review was conducted for each patient to ascertain treatment-related data. To stratify patients based on receipt of older and novel cytotoxics, the most commonly used cytotoxic agents were divided into two categories (A and B), noted in Table 1. Endocrine therapy was segregated into use of selective estrogen receptor modulators (SERMs), AIs, luteinizing hormone releasing hormone (LHRH) analogues, and fulvestrant. Patients who were treated with lapatinib or trastuzumab were coded as receiving HER2-directed therapy. Bone-directed therapy with intravenous bisphosphonates was coded separately for pamidronic and zoledronic acid. To examine temporal trends, the cohort was divided into two groups based on date of diagnosis: (1) between January 1, 1985 and December 31, 1994 (Period A), and (2) between January 1, 1995 and December 31, 2004 (Period B). Stratification into these two time periods was predicated on the approval of paclitaxel in 1994 – after this time, those agents listed into Category B (see Table 1) also gained FDA approval.

**Table 1:** Categorization of cytotoxic chemotherapies received during the study period

**Category A**	**Category B**
5-Fluorouracil	Capecitabine
Epirubicin	Docetaxel
Doxorubicin	Gemcitabine
Cyclophosphamide	Ixabepilone
Etoposide	Liposomal doxorubicin
Ifosfamide	*Nab-*paclitaxel
Methotrexate	Paclitaxel
Cisplatin	Vinorelbine
Carboplatin	

Novel agents were considered to be those approved since 1994 and are listed in Category B, while other available agents are included in Category A.

### Statistical analysis

Patients in each period were followed until the first of the following occurrences: death, date of last follow-up, or until 3 years after the end of the study period (i.e., December 31, 2008). Univariate analyses were performed by Cox regression to determine differences in survival for the following comparisons: period of diagnosis (Period A *v* Period B), age at diagnosis (≤ 50 *v* > 50), histology (inflammatory *v* invasive ductal), ER status (positive *v* negative), PR status (positive *v* negative), HER2 status (positive *v* negative), liver metastasis (yes *v* no), lung metastasis (yes *v* no), brain metastasis (yes *v* no), bone metastasis (yes *v* no), bone metastasis only (yes *v* no), supraclavicular metastasis (yes *v* no), soft tissue metastasis (yes *v* no), receipt of endocrine therapy (yes *v* no), receipt of SERMs (yes *v* no), receipt of AIs (yes *v* no), receipt of LHRH analogues (yes *v* no), receipt of fulvestrant (yes *v* no), receipt of chemotherapy (yes *v* no), type of chemotherapy rendered (receipt of cytotoxic agents from Category A *v* Categories A and B), receipt of HER2-directed therapy (yes *v* no), receipt of bisphosphonate therapy (yes *v* no), receipt of pamidronate acid (yes *v* no), and receipt of zoledronic acid (yes *v* no). Notably, with respect to ovarian suppression, only pharmacologic suppression with LHRH analogues was recorded; oophorectomy, ovarian ablation, and other surgical techniques were not captured. Variables bearing an association with survival with P<0.20 were incorporated in a multivariate analysis using stepwise regression. Variables with a P<0.10 were retained in the model. To examine the *a priori* hypothesis that advances in cytotoxic therapy have not resulted in cumulative improvements in survival, it was decided that the comparison of survival in patients receiving cytotoxic agents from Category A *v* Category A and B would be retained in the multivariate model, irrespective of the result on univariate analysis.

## Results

### Patient characteristics

Of 324 consecutive patients with a diagnosis of *de novo* MBC, 274 are included in the analysis. Reasons for exclusion included supraclavicular nodal disease only (n=29), misclassification of recurrent disease (n=15), and patient refusal of consent for research use of clinical information (n=6). Clinicopathologic and treatment-related data for all patients is summarized in Table 2. The median follow-up of survivors was 2.5 years for the overall population.

**Table 2:** Patient characteristics stratified by study period

**Characteristic**	Period A (n= 151)*		**Period B (n= 123)**		**P-value**
	**n**	**(%)**	**n**	**%**	
Median age, years	50		51		0.33
Histology					
Inflammatory	25	(17)	27	(22)	
Non-Inflammatory	126	(83)	96	(78)	0.59
Site of Metastasis					
Liver	28	(19)	45	(37)	0.30
Lung	45	(30)	39	(32)	0.24
Brain	4	(3)	10	(8)	0.72
Bone	84	(56)	82	(67)	0.008
Bone Only	37	(25)	31	(25)	0.52
Supraclavicular Node	55	(36)	34	(28)	0.20
Soft tissue	20	(13)	22	(18)	0.18
ER Status					
Positive	56	(63)	75	(68)	
Negative	33	(37)	34	(32)	0.01
PR Status					
Positive	43	(48)	54	(51)	
Negative	45	(52)	53	(49)	0.75
HER2 Status					
Positive	1	(50)	31	(39)	
Negative	1	(50)	32	(62)	0.71
Receipt of Endocrine therapy					
SERMs	82	(54)	52	(42)	0.0002
AI	19	(13)	46	(37)	0.72
LHRH	0	(0)	5	(4)	0.87
Fulvestrant	2	(1)	19	(15)	0.86
Receipt of Chemotherapy					
Category A**	85	(56)	15	(12)	
Category A + B	32	(21)	95	(77)	<0.0001
Receipt of HER2-directed therapy	2	(1)	19	(15)	0.17
Receipt of Bisphosphonates					
Pamidronic acid	8	(5)	37	(30)	0.0007
Zoledronic acid	3	(2)	35	(28)	0.04

Percentages reflect the proportion of patients in each subgroup relative to the total number of patients in the respective period (i.e., Period A or Period B), except for ER, PR, and HER2 status. For these variables, percentages reflect the proportion of patients in each subgroup relative to the total number of patients in the respective period for whom pathologic data is available. P-values determined using the Chi-square test.

* Period A encompasses patients diagnosed between January 1, 1985 and December 31, 1994. Period B encompasses patients diagnosed between January 1, 1995 and December 31, 2004.

** See Table 1 for delineation of cytotoxic agents within Category A and Category B.

### Univariate analyses

Univariate analyses of survival by clinicopathologic predictors are presented in Table 3. Patients diagnosed during period B had a slightly longer median survival as compared to patients treated during period A, although this difference was not statistically significant (3.0 years vs 2.5 years, respectively; P=0.10). Among the clinicopathologic factors assessed, only ER status was significantly associated with improved survival (hazard ratio [HR] 0.69, 95%CI 0.50-0.96; P=0.03). On the other hand, with treatment-related variables, multiple significant associations with survival were observed (Table 4). Treatment with endocrine therapy was associated with an improvement in survival (HR=0.60, 95%CI 0.47-0.77; P<0.0001), and within this category, treatment with SERMs, AIs, and fulvestrant were each associated with improvements in survival (P=0.007, P<0.0001, and P=0.0004, respectively). Although receipt of chemotherapy approached significance as a predictor of survival (HR 0.76, 95%CI 0.57-1.02; P=0.06), there was no significant difference in survival among patients who received agents exclusively from Category A as opposed to both Category A and B (P=0.13). In the current analysis, patients receiving HER2-directed agents comprised a relatively small subset (n=21), challenging inferences in this subset. Lastly, bisphosphonate therapy was associated with improved survival (HR 0.70, 95%CI 0.52-0.96; P=0.02), and within this category, receipt of zoledronic acid was associated with the same (HR 0.50, 95%CI 0.32-0.77; P=0.002).

**Table 3:** Univariate analyses comparing survival in patient subgroups stratified by clinicopathologic characteristics

**Characteristic**	**Hazard ratio**	**95%CI**	**P-value**
Date of diagnosis			
Period A	1.00	reference	0.10
Period B	0.95	0.92 – 1.00	
Age (years)			
≤ 50	1.00	reference	0.06
> 50	1.27	1.00 – 1.63	
Histology			
Non-Inflammatory	1.00	reference	0.21
Inflammatory	1.22	0.88 – 1.69	
ER Status			
Negative	1.00	reference	0.03
Positive	0.69	0.50 – 0.96	
PR Status			
Negative	1.00	reference	0.17
Positive	0.8	0.59 – 1.09	
HER2 Status			
Negative	1.00	reference	0.08
Positive	0.55	0.28 – 1.08	
Liver Mets			
No	1.00	reference	0.60
Yes	1.08	0.80 – 1.44	
Lung Mets			
No	1.00	reference	0.10
Yes	1.25	0.95 – 1.65	
Brain Mets			
No	1.00	reference	0.22
Yes	1.43	0.80 – 2.56	
Bone Mets			
No	1.00	reference	0.06
Yes	1.29	0.98 – 1.70	
Bone Only Mets			
No	1.00	reference	0.23
Yes	0.83	0.62 – 1.12	
Supraclavicular Node Mets			
No	1.00	reference	0.56
Yes	0.92	0.69 – 1.22	
Soft Tissue Mets			
No	1.00	reference	0.53
Yes	1.11	0.78 – 1.59	

**Table 4:** Univariate analyses comparing survival in patient groups stratified by treatment-related characteristics

**Characteristic**	**Hazard ratio**	**95%CI**	**P-value**
Endocrine Therapy			
No	1.00	reference	<0.0001
Any	0.60	0.47 – 0.77	
SERM			
No	1.00	reference	0.007
Yes	0.63	0.48 – 0.82	
AI			
No	1.00	reference	<.0001
Yes	0.47	0.34 – 0.66	
LHRH			
No	1.00	reference	0.75
Yes	0.85	0.31 – 2.30	
Fulvestrant			
No	1.00	reference	0.0004
Yes	0.29	0.15 – 0.57	
Chemotherapy			
No	1.00	reference	0.06
Yes	0.76	0.57 – 1.02	
Category			
A	1.00	reference	
A+B	0.8	0.60 – 1.07	0.13
HER2-Directed Therapy			
No	1.00	reference	0.15
Yes	0.69	0.41 – 1.14	
Bisphosphonate Therapy			
No	1.00	reference	0.02
Yes	0.70	0.52 – 0.96	
Pamidronic acid			
No	1.00	reference	0.44
Yes	0.87	0.62 – 1.23	
Zoledronic acid			
No	1.00	reference	0.002
Yes	0.50	0.32 – 0.77	

### Stepwise multivariate analysis

In order to account for the possible confounding issues, the estimator for the variable of interest (Category A *v* [Category A and B]) was examined. A model with all the terms was created and then each variable was taken out and the variable of interest’s estimator was evaluated. Ultimately age, receipt of AI, and receipt of zoledronic acid were found to be independent significant predictors of survival; while category (i.e., Category A *v* [Category A + B]) was not. The adjusted variables are listed in order of significance in Table 5.

**Table 5:** Multivariate analysis comparing survival in subgroups of patients with *de novo *MBC stratified by both clinicopathologic and treatment-related characteristics

**Characteristic**	**Hazard ratio**	**95% CI**	**P-value**
Category			
A	1.00	reference	0.76
A + B	1.04	0.77 – 1.40	
Age			
<50	1.00	reference	0.0008
≥50	1.61	1.23 – 2.20	
AI			
No	1.00	reference	0.0008
Yes	0.50	0.34 – 0.75	
Zoledronic acid			
No	1.00	reference	0.005
Yes	0.45	0.26 – 0.79	

## Discussion

The impact of systemic therapy on the survival of women with metastatic breast cancer over time has been addressed by seven different investigators and these results are summarized in Table 6. All studies suggest that modest improvements in overall survival have been observed in recent years. An improvement in overall survival advantage is most pronounced in the trials where a higher percentage of women have hormone receptor-positive disease. We also observed a survival advantage for women with hormone receptor-positive disease and those who received an aromatase inhibitor. These observations are consistent with the published literature which demonstrates that a third generation aromatase inhibitor prolongs the survival of women with MBC compared with a progestational agent [11,12]. 

**Table 6:** Summary of trials addressing systemic therapy in advanced breast cancer

**Author, year**	**Institution(s)/ Database**	**Population**	**Time intervals**	**Comments**
Andre, 2004 [5]	France (3 institutions)	724 women *de novo* metastatic breast cancer	1987-1993	Excluded supraclavicular lymph node only patients. Improved 3 year overall survival (OS; 44% vs 27%) was seen in the more recent time period.
			1994-2000	
Giordano, 2004 [9]	MD Anderson Cancer Center	834 women with recurrence after adjuvant doxorubicin	1974-1979	Included patients with locally recurrent disease. Predictors of improved overall survival included stage at diagnosis, interval disease-free survival, and dominant metastatic site.
			1980-1984	
			1985-1989	
			1990-1994	
			1995-2000	
Chia, 2007 [6]	British Columbia Cancer Agency	2150 women with both recurrent and *de novo* metastatic breast cancer	1991-1992	An improvement in overall survival was noted over time, primarily in those patients that were hormone-receptor positive.
			1994-1995	
			1997-1998	
			1999-2001	
Dawood, 2008 [13]	Surveillance, Epidemiology, and End Results Registry	15,438 women with both recurrent and *de novo* metastatic breast cancer	1988-1993	There was a modest improvement in overall survival, associated with patients with hormone-receptor positive disease. Outcomes appeared to be worse in African American women.
			1994-1998	
			1999-2002	
			2003-2006	
Dafni, 2009 [14]	Hellenic Cooperative Group	1361 enrolled on trials for treatment of recurrent and *de novo* metastatic breast cancer	1991-1994	Trastuzumab used in 24% and 30% of patients in the two most recent time intervals.
			1995-1998	
			1999-2003	
Ruiterkamp, 2011 [10]	Netherlands	8031 women with both recurrent and *de novo* metastatic breast cancer	1995-1999	10% of patients in third time interval received targeted therapy.
			2000-2004	
			2005-2008	
Pal, 2011	City of Hope	274 *de novo* metastatic breast cancer only	1985-1994	Overall survival improved for those receiving endocrine therapy, but no apparent overall survival benefit with newer chemotherapeutic agents.
			1995-2004	

The Hellenic Trialists compared the overall survival for women enrolled in 10 different treatment trials for MBC over a 15 year time interval and reported improving survivals over time [7]. This Greek series is confounded by the inclusion of women on trastuzumab-containing regimens since 1999, a treatment that is unquestionably associated with an improved overall survival. In our series, the number of women receiving trastuzumab-containing chemotherapy was too small to demonstrate a survival advantage.

In our study and that of Andre, the trials included only patients who presented with *de novo* metastatic disease and reported an overall improvement in survival [5]. However, the survival advantage was largely attributed to benefits from endocrine therapy. We found no advantage to the use of the newer chemotherapeutic agents and Andre found no survival advantage for women with hormone receptor-negative disease [5].

We uniquely identified that the use of intravenous zoledronate was predictive of an improved survival. Given the lack of data supporting a survival advantage for bone-directed therapies in metastatic disease, we hypothesize that the improved survival observed is indicative of improved palliative care.

All the published series are retrospective reviews and the results are confounded by improvements in radiologic imaging, changes in the staging system, and potential for lead time bias. Some of the series included women with locally recurrent disease, a pattern of recurrence that is associated with a more favorable outcome. Only our study and that of Andre included only patients with *de* novo metastatic disease [5]. It remains uncertain if the natural history of *de novo* metastatic disease differs meaningfully from recurrent disease after treatment of early stage cancer. We are currently reviewing the outcome using the same analysis employed here to assess the impact of newer systemic therapy in women with recurrent MBC. We anticipate that this will provide additional hypothesis-generating data.

## Conclusions

In reviewing the available literature, it appears that advances in endocrine therapy and trastuzumab are largely responsible for overall survival improvements in the treatment of metastatic breast cancer. Improvements in endocrine therapy and the use of trastuzumab appear to have improved the survival of women with advanced breast cancer. The impact of conventional chemotherapy on survival appears to be minimal in the context of our retrospective cohort; perhaps these findings warrant more definitive exploration through the formation of multi-institution databases. These multi-institutional databases should include ideally include the same granularity of data as in our analysis and other, and should also capture surgical and radiation data to infer the benefits rendered from these modalities. Findings from such efforts could surely supplant the existent studies which draw from limited single-institution experiences.

## Competing interests

The authors declare that they have no competing interests.

## Authors’ contributions

SKP, MD, RAN, SO, and JM participated in the conception and design of the project. SKP, MD, RAN, SO, SW, LK, and JM participated in data collection. SKP, MD, RAN and JM participated in data analysis. SKP, MD, RAN, SO, JH, SW, LK and JM participated in the writing of the manuscript, and all authors had final approval of the submitted manuscript.

## Pre-publication history

The pre-publication history for this paper can be accessed here:

http://www.biomedcentral.com/1471-2407/12/435/prepub
